# Corticobulbar activity in healthy humans and Parkinson’s disease: a study protocol for a novel biomarker of motivational arousal

**DOI:** 10.3389/fpsyg.2025.1573534

**Published:** 2025-07-17

**Authors:** Francesca Ferraioli, Francesco Tomaiuolo, Alessandra M. Falzone, Angelo Labate, Salvatore M. Cardali, Simona Massimino, Fabrizio Esposito, Antonino Germanò, Luigi Trojano, Carmelo Mario Vicario

**Affiliations:** ^1^Laboratory of Cognitive and Social Neuroscience, Department of Cognitive Science (COSPECS), University of Messina, Messina, Italy; ^2^Department of Clinical and Experimental Sciences Biomedical, Dental and Imaging Morphological and Functional, Messina, Italy; ^3^Neurophysiology and Movement Disorders Unit, University of Messina, Messina, Italy; ^4^Department of Neurosurgery, Azienda Ospedaliera Papardo, University of Messina, Messina, Italy; ^5^Department of Advanced Medical and Surgical Sciences, MRI Research Center, Università degli Studi della Campania Luigi Vanvitelli, Naples, Italy; ^6^Department of Psychology, University of Campania Luigi Vanvitelli, Caserta, Italy

**Keywords:** corticobulbar excitability, TMS, virtual reality, Parkinson’s disease, submental EMG, motivational arousal, methods and protocols

## Abstract

**Background:**

The corticobulbar (CB) tract connects the primary motor cortex to oral and facial effectors and may contribute to affective-motivational processes through its interactions with dopaminergic circuits. Prior studies have shown that excitability in the tongue motor cortex (tM1), as well as surface electromyographic (sEMG) activity of submental muscles (SbM), is modulated by hedonic and aversive stimuli. These findings suggest a potential role for the CB system as a physiological interface between motivational states and motor expression.

**Objectives:**

This protocol explores whether CB excitability can serve as an indirect marker of motivational arousal in both healthy individuals and patients with Parkinson’s disease (PD), a condition marked by dopaminergic dysfunction.

**Methods:**

Study 1 protocol consists in a virtual reality-based shopping task where healthy adults (*n* = 100) are asked to do different food shipping based on motivational-affective value, while their SbM EMG activity is recorded. Study 2 assesses CB excitability via SbM motor evoked potentials (MEPs) in PD patients (*n* = 15) before and after a 3-month period of dopaminergic therapy (levodopa), and across ON/OFF medication states. The study design includes a control group (*n* = 15) tested twice, after a 3-month period, without any drug or placebo administration. Behavioral and self-report measures related to motivation and reward sensitivity are also included in both studies.

**Conclusion:**

This protocol combines advanced neurophysiological techniques with innovative experimental paradigms to investigate CB tract cortical excitability linkage with reward-related processing. By integrating neurophysiological, behavioral, and pharmacological measures, this protocol aims to clarify whether CB excitability reflects motivational and dopaminergic states. Findings may contribute to identifying non-invasive biomarkers of motivational functioning in both clinical and normative populations.

## 1 Introduction

Dopaminergic pathways are fundamental to reward, motivation, appetite and affects, playing a pivotal role in homeostasis and adaptive behaviors (Bressan and Crippa, 2005; Volkow et al., 2011; Baik, 2013; Panksepp and Moskal, 2013; Volkow et al., 2017; Speranza et al., 2021). Dysfunction in these pathways is implicated in various neurological and psychiatric disorders, including Parkinson’s Disease (PD), where dopaminergic neurons degeneration significant affects motor, cognitive, and affective functions (Bressan and Crippa, 2005; Marsden, 2006; Baik, 2013; Speranza et al., 2021), and disrupts their finely tuned balance, contributing to PD ‘s complex clinical profile (Lees et al., 2009).

Motivational arousal refers to the neurophysiological readiness to act in response to rewarding stimuli. It involves the capacity to evaluate incentive salience and to mobilize resources toward goal-directed behaviors (Berridge, 2004; Salamone and Correa, 2012). In this framework, we explore whether corticobulbar excitability may serve as an index of such arousal, reflecting variations in reward sensitivity and motivational engagement.

The corticobulbar (CB) tract, connecting the primary motor cortex to muscles controlling swallowing, tongue movement, and facial expressions offers a promising avenue for investigating dopaminergic processes (Love and Webb, 1992; Gonzalez-Fernandez et al., 2018; Wilmskoetter et al., 2020). Specifically, the tongue motor cortex (tM1), whose descending projections form part of the CB tract, has emerged as a critical node within the interface between motor and affective systems. CB tract function is closely linked to dopaminergic activity, particularly in mesolimbic pathways associated with reward and motivational arousal (Skitek et al., 1999; Stanford et al., 2003; Nuckolls et al., 2012). Altered CB excitability in PD patients, often manifesting speech impairments, reduced tongue endurance, and swallowing dysfunction, underscores the potential of this tract as a biomarker for disease progression and motivational deficits (Potulska et al., 2003; De Letter et al., 2007). Taken together, these findings suggest a functional circuit linking tM1 cortical excitability, descending corticobulbar output, and submental muscle response, modulated by dopaminergic signaling and relevant for assessing motivational arousal.

Uniquely positioned at the intersection of motor control and reward processing networks (Vicario et al., 2022), the CB tract’s motor-cortical representation receives and transmits information from key reward system regions, such as the amygdala, orbitofrontal cortex (OFC), anterior cingulate cortex (ACC), and striatum, in non-human primates (Alipour et al., 2002; Passingham, 2021). This rich connectivity suggests the CB tract as a valuable pathway for assessing and measuring reward-related processes, offering potential insights into neurobiological mechanisms underlying motivational deficits in clinical conditions like PD, eating disorders, and addiction.

However, PD-related dopaminergic neuron degeneration extends beyond the motor system, affecting key reward processing areas such as the OFC, ventral striatum, and amygdala (Kringelbach et al., 2003; O’Doherty, 2004; Kringelbach, 2005). Alterations in dopaminergic signaling, particularly within striatal circuits, significantly alter reward processing and motivational arousal. For example, reduced striatal D2 receptor availability is linked to compulsive food intake in obese rodents (Johnson and Kenny, 2010) and to decreased OFC and ACC metabolic activity in obese humans (Tremblay and Schultz, 1999). Similar dopaminergic dysfunctions is implicated in the reward sensitivity and food intake observed in Anorexia Nervosa (AN) (Kaye et al., 2009). These findings underscore the importance of investigating dopaminergic modulation of CB tract function to understand its role in linking reward dysregulation to motivational deficits across neurological and psychiatric conditions, as detailed in a recent perspective paper.

Transcranial magnetic stimulation (TMS) and surface electromyography (sEMG), enables direct assessment of the functional output of CB tract excitability. TMS-evoked motor evoked potentials (MEPs) recorded via sEMG sensitively index cortical excitability and motor pathway integrity (Nardone et al., 2015; Spampinato et al., 2023); spontaneous sEMG measures muscle activity, providing complementary information on neural connectivity and muscle function (Enders and Nigg, 2016; Balbinot et al., 2021). Previous studies using non-invasive brain stimulation (NIBS) techniques suggest that CB excitability—measured via MEPs in tongue motor cortex (tM1), which are innervated via the CB tract—is modulated by motivationally relevant states. For example, changes in TMS-induced MEPs have been reported in response to nicotine craving in abstinent smokers (Vicario et al., 2014), core and social disgust (Vicario et al., 2017; Vicario et al., 2022), and appetite (Vicario et al., 2020). Although these findings do not directly isolate CB tract excitability, they provide converging evidence that tM1 activity and its corticobulbar projections may reflect dopaminergic and motivational modulation.

The submental musculature (SbM), crucial for tongue movements and swallowing, is ideally suited for such investigations due to its role in hedonic and motivational behaviors, such as feeding and speech (Ertekin, 2011; Sato et al., 2021). Notably, animal studies show SbM dopaminergic connectivity, underscoring its relevance in assessing the reward system function (Enomoto et al., 2011; Chen et al., 2019). Levodopa, a primary PD treatment, modulates CB-related symptoms, improving tongue movement and swallowing (De Letter et al., 2007). The present protocol study, analyzing both the effects of levodopa on SbM MEPs elicited via TMS, and spontaneous SbM EMG activity in healthy individuals, offers insight into how dopaminergic modulation shapes corticobulbar output in PD, as well as natural variations reflected in motivational arousal along the same pathway.

Motivational arousal is closely linked to dopaminergic function and represents a key dimension in both normative behavior and clinical disorders. It is traditionally assessed through self-report or behavioral paradigms, but emerging evidence suggests that physiological indices such as CB excitability and submental sEMG may capture the somatic expression of affective-motivational states, particularly in response to hedonic stimuli.

Building on this literature, the present protocol explores an integrated way to investigate whether CB excitability reflects motivational arousal across two complementary studies.

In Study 1, we hypothesize that sEMG activity of the SbM will vary in function of different motivational valence shopping-scenario (Daily, Hedonic, Dislike), in a sample of 100 healthy participants. We expect SbM sEMG activity will increase/decrease with respect to the three motivational scenarios, reflecting implicit motivational engagement.

In Study 2, we hypothesize that CB excitability, indexed via TMS-evoked potentials, will be sensitive to ON/OFF medication states in a sample of 15 PD patients comparing 15 matched-age controls. Specifically, we will examine variations in motor evoked potential (MEP) amplitude, latency, and resting motor threshold across conditions.

Taken together, the two described protocols aim to assess the same neurophysiological system across healthy and clinical populations. Study 1 is designed to characterize normative responses in healthy individuals, while Study 2 tests the same marker—SbM activity—under dopaminergic modulation in Parkinson’s disease patients. This integrated approach may provide converging evidence for the validity of assessing corticobulbar excitability as a non-invasive biomarker of motivational processing.

Overall, this research aims at establishing a comprehensive framework linking CB tract function dopaminergic activity. Utilizing advanced neurophysiological tools with innovative experimental designs, these studies will investigate the potential of SbM activity as both a clinical and normative relevant biomarker for motivational arousal deficits and dopaminergic dysfunction, informing the development of innovative interventions, as diagnostic and therapeutic strategies for neurological and psychiatric disorders.

## 2 Methods and analysis

The following section outlines the methodological and analytical framework of the protocol. It is organized into two main blocks corresponding to the two studies: Study 1, where healthy participants are required to perform a virtual reality shopping task and Study 2, where patients with Parkinson’s Disease undergo TMS stimulation. Each study is structured into four subsections detailing the study design, participant selection, experimental procedures, and data analysis plan.

### 2.1 Study 1 on healthy participants: spontaneous sEMG during motivational VR task

#### 2.1.1 Participant selection

For the first study we will recruit 100 healthy adults among university students and community volunteers from Messina University. The sample will be balanced for gender to ensure representative data collection. The target sample size of 100 participants was determined based on previous studies using similar immersive virtual paradigms in response to motivationally salient stimuli (Melendrez-Ruiz et al., 2022; Ferraioli and Vicario, 2024). This sample allows for sufficient sensitivity to detect within-subject differences in physiological and behavioral responses across conditions, while accounting for inter-individual variability.

Inclusion Criteria: Participants aged 18 to 45 years, with normal or corrected-to-normal vision, and no reported history of neurological or psychiatric disorders.

Exclusion Criteria: Individuals will be excluded in the presence of any contraindications to Virtual Reality (VR) exposure, such as a history of severe motion sickness, and of significant visual impairments not corrected by lenses. Participants with current use of medications affecting neural activity or those unable to provide informed consent will also be excluded.

Recruitment and Consent: Recruitment will be conducted through digital advertisements on social media platforms, posters in local academic institutions and presentation of the experiment during courses at COSPECS dept. of Messina University. Interested participants will receive detailed study information and sign a digital informed consent form before enrollment. A single blind procedure will be applied during data collection. Ethical approval for this protocol (amendment to Protocol No: COSPECS_03_2022) has been granted by the Ethics Committee of the COSPECS Department at the University of Messina.

#### 2.1.2 Interventional methods

##### 2.1.2.1 Study design

**Figure 1:**
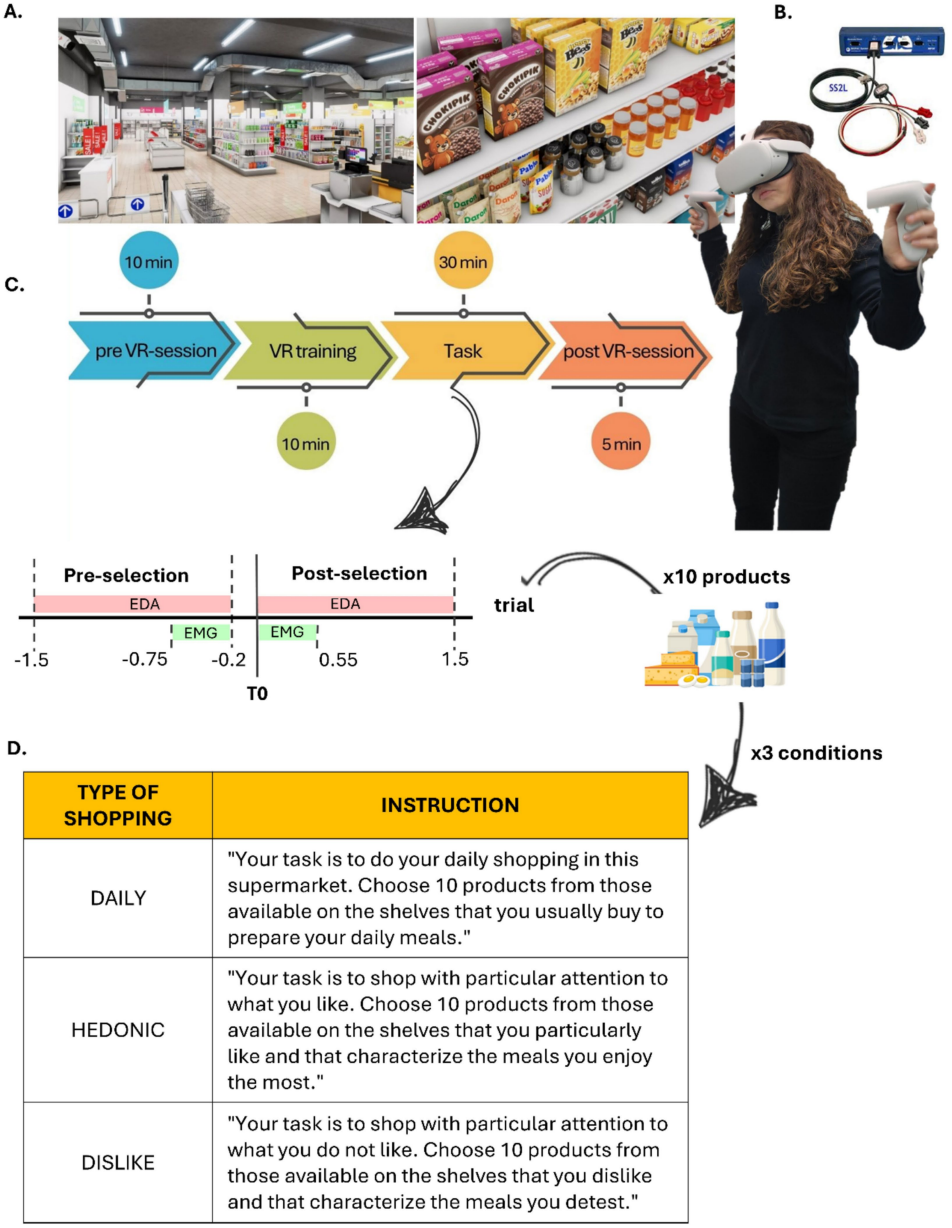
Experimental set-up and timeline Study 1. **(A)** Virtual Environment: Images of the virtual supermarket used, obtained from the Unity 3D store. **(B)** Technical Equipment: Oculus Quest 2 for virtual navigation and interaction, BIOPAC MP36 for electrophysiological recordings. **(C)** Timeline of experimental phases. **(D)** Table with instruction received by participants for each experimental condition.

**Study 1** adopts a within-subject design to investigate spontaneous submental muscle activity (sEMG) in response to motivationally salient stimuli within an ecologically valid VR shopping environment. By integrating behavioral and electrophysiological data, this study aims to establish SbM EMG as a reliable biomarker for hedonic/disgusting experiences and sensory preferences in a controlled and ecologically valid environment, in line with pilot investigations (Ferraioli and Vicario, 2024). The experimental setup is designed to mimic real-world consumer scenarios while maintaining the rigor of laboratory-based measurements. All participants will be exposed to three motivationally distinct shopping scenarios: Daily Needs, Hedonic Reward, and Dislike/Aversive. Each scenario requires the selection of 10 products from a standardized pool. The conditions are presented in counterbalanced order across participants. The given instruction for the three conditions is summarized in Figure 1 (table below). Although the VR supermarket is fully navigable, participants are instructed during the training phase to focus only on selected aisles. Each scenario lasts approximately 10 min, preceded by a 1-min baseline exploration and followed by product evaluations, resulting in a total task time of approximately 30 min.

##### 2.1.2.2 Experimental procedure

Participants are immersed in a VR supermarket built, obtained and modified from Unity 3D assets and navigated via Oculus Quest 2 headset using hand-held controllers, which visually represent the participant’s hands. Navigation is limited to selected aisles covering a range of product categories (e.g., produce, dairy, breakfast, frozen foods, deli, and beverages) to reduce session length and minimize motion sickness. Interaction is facilitated via hand-held controllers, represented by real hands in the VR environment, providing realistic and immersive experience, as shown in Figures 1A,B.

The experimental task adapted from Melendrez-Ruiz et al. (2022), consists of a single session where participants are instructed to select 10 products for each of the three following experimental conditions:

1. Daily Selection: Products typically purchased for daily meals;
2. Hedonic Selection: Products participants particularly enjoy or find appealing;
3. Disliked Selection: Products they actively avoid or find unappealing.

During the task, surface EMG of the SbM will be recorded to capture physiological responses associated with food preferences. EMG signals will be collected using pre-gelled, self-adhesive Ag/AgCl electrodes, positioned according to standardized guidelines and previous studies (Fridlund and Cacioppo, 1986; Sato et al., 2021; Ferraioli and Vicario, 2024). Signals will be sampled at 1,000 Hz using a BIOPAC MP200 system; the alignment between the timings of VR events and electrophysiological responses will be done by customized Matlab script in the post-processing. Signal preprocessing is performed in MATLAB using: 50 Hz notch filtering, 25–400 Hz band-pass filtering (4th-order Butterworth), full-wave rectification, and Root Mean Square smoothing with a 50 ms sliding window. Signals are normalized to a 1-min pre-task baseline. T0 (product selection onset) is determined using a custom script that allows the experimenter to mark the start of the grasping action within the VR system. For each trial, EMG activity is segmented into two windows: (1) pre-selection phase: −550 to −200 ms before T0, and (2) post-selection phase: T0 to +550 ms. RMS-rectified values are calculated for both windows and used for subsequent analyses.

In addition to EMG data, during the experimental session, participants will be asked to rate each selected product 5 s after selection on a 10-point Likert scale assessing the following questions:

Pleasantness: How much they like the product.
Healthiness: Perceived nutritional value.
Desire to consume: Likelihood of consuming the product.
Purchase probability: Likelihood of purchasing the product.

During the whole experimental session, we also collect behavioral measurements. Before the experimental session participants will fill the Eating Attitudes Test-26 (EAT-26) (Garner et al., 2013), a standardized instrument used to assess potential eating disorders and abnormal eating behaviors. It includes 26 items scored on a 6-point Likert scale and is subdivided into three subscales: dieting, bulimia and food preoccupation, and oral control. Higher scores indicate more problematic eating attitudes. BMI will be further calculated using self-reported height and weight, as well as reported appetite score (10-point Likert’s scale). Participants will complete additional questionnaires to assess impulsivity and reward sensitivity (MCQ) (Kaplan et al., 2016), chronotype (MEQ-SA) (Terman and Terman, 2005). Further, after the VR session, they also rate the feeling of presence, that is the perceived immersion in the VR environment experience (Presence Questionnaire, PQ) (Witmer and Singer, 1998).

#### 2.1.3 Data analysis

Descriptive statistics will summarize EMG data and subjective ratings across conditions. Repeated measures ANOVA will test for differences in SbM activation across the three motivational contexts. If normality assumptions are violated, non-parametric alternatives (e.g., Friedman tests) will be used. Pairwise comparisons will be Bonferroni-corrected.

Correlational analyses (Pearson or Spearman depending on distribution) will examine associations between EMG activation and subjective measures such as product ratings, BMI, appetite, and impulsivity scores.

Finally, linear mixed-effects models will be constructed to predict participants’ preference and purchase probability ratings based on sEMG amplitude. Fixed effects will include motivational condition and EMG window (baseline/response), while random intercepts will account for inter-individual variability. MCQ scores will be included as covariates to assess the influence of delay discounting traits on hedonic preference formation, in line with Ferraioli (2024).

##### 2.1.3.1 Expected results

Based on previous findings and pilot data (Ferraioli, 2024; Ferraioli and Vicario, 2024), we expect submental EMG activation to significantly differ across motivational conditions, with higher amplitudes in the Hedonic condition compared to both Daily and Dislike, particularly in the pre-selection phase (−550 to −200 ms before T0).

Correlational analyses are expected to reveal positive associations between EMG amplitude and subjective ratings of pleasantness and purchase probability; further we expect to find correlation also with individual differences assessed by questionnaires.

Moreover, the linear mixed-effects model based on pre-selection EMG activity is expected to predict individual ratings of pleasantness. Furthermore, we will add MCQ as fixed effect hypothesizing that individual differences in delay discounting (as measured by the MCQ) will moderate conditions effect: participants with lower discounting rates (i.e., more future-oriented decision-makers) are expected to show stronger predictive coupling between EMG amplitude and subjective valuation, suggesting greater sensitivity to the motivational salience of appetitive stimuli.

### 2.2 Study 2 on Parkinson’s patients: TMS-evoked MEPs from submental muscle

#### 2.2.1 Patients selection

Study 2 will include a total of 30 participants: 15 individuals diagnosed with idiopathic Parkinson’s Disease (PD) and 15 age-and gender-matched healthy controls. Patients will be recruited from local neurology clinics and hospitals (Policlinic of Messina), affiliated with the University of Messina.

A-priori power analysis was conducted using the “pwr” package in R, assuming a large effect size (Cohen’s *f* = 0.40) based on previous studies reporting significant increases/decreases in MEP amplitude following reward-related manipulations (Vicario et al., 2014, 2017; Vicario et al., 2015; Vicario et al., 2022), as well as evidence from animal models demonstrating dopaminergic modulation of gambling-like behaviors (Cocker et al., 2017). With a significance level of *α* = 0.05 and statistical power (1 − *β*) = 0.80, the estimated total sample size was 52 participants. However, considering the exploratory nature of this protocol and the practical constraints in recruiting PD patients eligible for OFF-medication testing, we opted to recruit a reduced sample of 30 participants (15 PD patients and 15 matched controls).

*De novo* patients will be prioritized for inclusion. However, if recruitment proves insufficient, the sample will be extended to include patients undergoing an overnight washout (≥12 h) to obtain a reliable OFF-medication condition. In the latter case, participants on stable levodopa therapy will be asked to temporarily suspend medication for at least 12 h (overnight withdrawal) to ensure a clinically meaningful OFF condition, as widely adopted in PD research. The ON-medication session will take place on a different day, after the patient’s regular dose of levodopa has taken effect, following their standard medication schedule.

Inclusion criteria (PD group): 15 Patients and 15 healthy controls aged 45–65 years. Patients with PD have to be classified within early-stage PD by Movement Disorder Society - Unified Parkinson’s Disease Rating Scale (MDS-UPDRS, Italian version; Goetz et al., 2008), and should be capable of providing their informed consent.

Exclusion criteria: Patients with atypical Parkinsonism, severe motor complications, or contraindications to TMS (e.g., presence of metallic implants or epilepsy), according to standard guidelines (Wassermann, 1998; Rossi et al., 2021), will be excluded. Cognitive impairments precluding informed consent or participation in the tasks will also result in exclusion.

Recruitment and Consent: Recruitment will involve direct referrals from neurologists and advertisements in clinic waiting areas. Patients will receive detailed explanations of the study, and written informed consent will be obtained. Ethical approval (protocol No: CEL 12–24) has been secured through the relevant hospital boards. Healthy control participants will be recruited from the local community using comparable inclusion/exclusion criteria, excluding any history of neurological or psychiatric disorders. All participants will provide their written informed consent. The protocol has received ethical approval from the COSPECS Ethics Committee at the University of Messina (amendment to protocol No: COSPECS_03_2022).

#### 2.2.2 Interventional methods

##### 2.2.2.1 Study design

Study 2 adopts a mixed design combining within-subject comparisons in the PD group and between-group comparisons with healthy controls. The primary objective is to assess whether CB excitability, as indexed by TMS-evoked MEPs from the SbM, is modulated by dopaminergic state in Parkinson’s Disease.

Each participant with PD will complete two experimental sessions:

1. OFF-medication condition: following a minimum 12-h withdrawal from levodopa.
2. ON-medication condition: performed on a separate day, after the intake and expected effect of their routine levodopa dose.

Healthy controls will be tested twice (after a 3-month period) to provide normative data for comparison. This design enables evaluation of both the effects of levodopa in patients with PD and the differences between PD and healthy populations. Further, to assess the specificity of dopaminergic modulation on the corticobulbar pathway, MEPs will be recorded not only from the submental muscle but also from a control muscle (the extensor carpi radialis, ECR). This allows for distinguishing region-specific changes in cortical excitability from general motor system fluctuations.

##### 2.2.2.2 Experimental procedure

Participants will be seated comfortably in an adjustable armchair and instructed to remain relaxed throughout the session. sEMG will be recorded from the SbM and from a control muscle (ECR), using BIOPAC MP200 system, to assess the specificity of corticobulbar excitability changes. Bipolar pre-gelled Ag/AgCl electrodes (1 cm inter-electrode spacing) will be placed according to standardized guidelines, ensuring low skin impedance.

**Figure 2:**
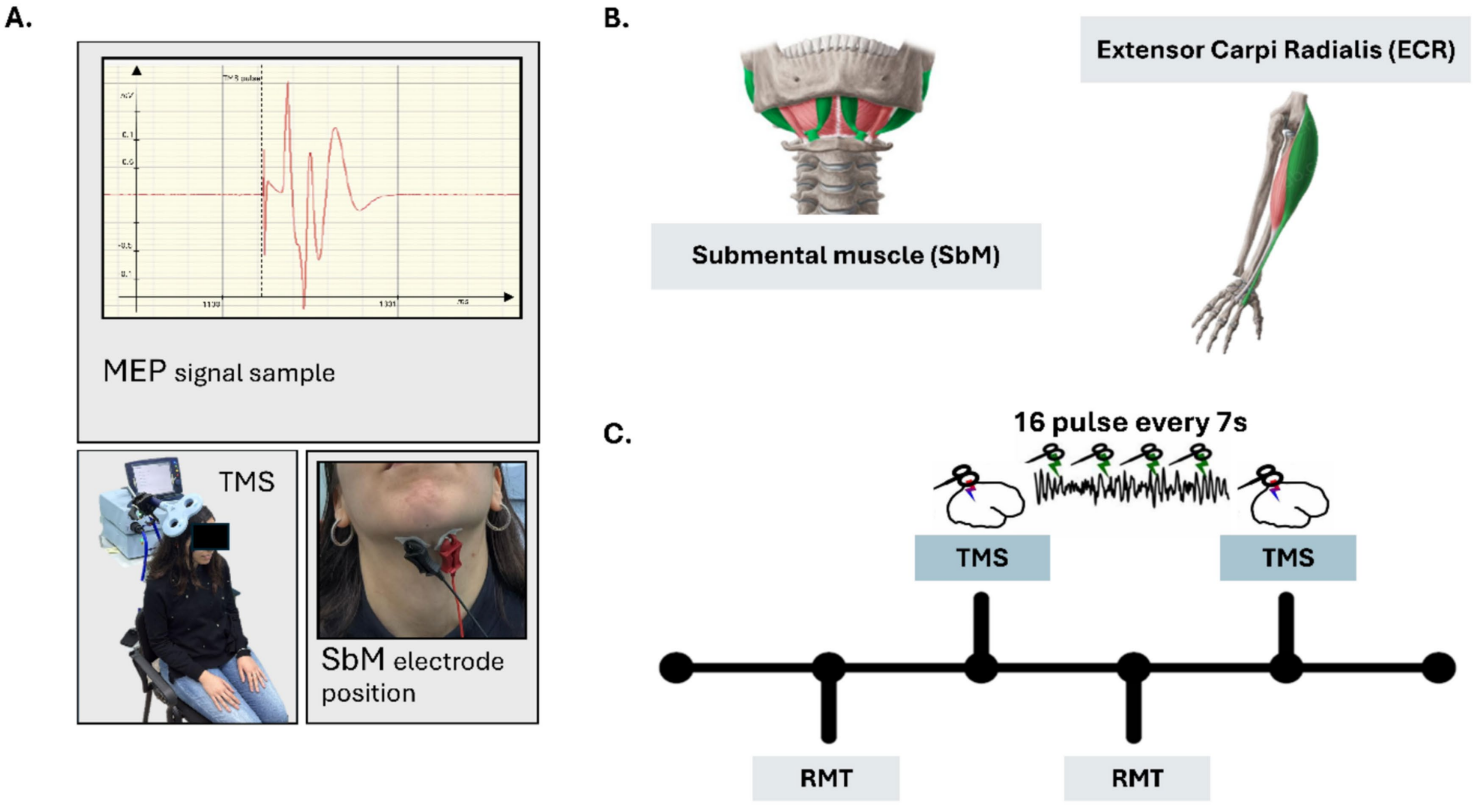
Experimental Set-up and timeline Study 2. **(A)** An example of the experimental setup for recording MEPs from M1 cortex of the SBM. **(B)** Graphical representation of the target muscle (SbM) and the control muscle (ECR); **(C)** The timeline of the experimental session. The lower section illustrates the initial phases, which involve determining the resting motor threshold (RMT) for both the target muscle (SbM) and the control muscle (ECR). The upper section shows the subsequent stimulation blocks, during which TMS is applied to each muscle in a counterbalanced order across participants.

TMS will be delivered over the SbM motor area using a figure-of-eight coil connected to the Magstim Super-Rapid stimulator. The coil will be placed tangentially over the scalp at a ~ 45° angle to the midline to induce posterior–anterior current flow. The optimal site for evoking consistent MEPs in the SbM will be identified individually and marked on the scalp with a non-permanent marker to ensure reproducibility between sessions. The resting motor threshold (RMT) will be determined separately for both SbM and ECR, defined as the minimum stimulator intensity required to evoke MEPs ≥ 50 μV peak-to-peak amplitude in at least 5 out of 10 trials. For the experimental phase, 16 single TMS pulses will be delivered at 120% of RMT with inter-stimulus intervals randomly jittered between 5 and 7 s to prevent habituation. Orientation and stimulation parameters will be optimized based on previous work (e.g., Vicario et al., 2014). An example of MEPs temporal dynamics is shown in Figure 2A.

All sessions will last approximately ~ 60 min including setup, RMT determination, and MEP acquisition. The ON and OFF sessions for PD patients will be scheduled at least 72 h apart, at the same time of day to minimize circadian effects. The healthy control group will undergo the same procedures in two sessions, spaced 3 months apart.

Behavioral measures will complement physiological data that will be analyzed along with electrophysiological indices. Participants will complete self-report questionnaires, such as Parkinson’s Disease Fatigue Scale (PFS-16; Italian version, Siciliano et al., 2019) and Hospital Anxiety and Depression Scale (HADS; Italian version, Costantini et al., 1999).

#### 2.2.3 Data analyses

MEPs will be analyzed offline from both the SbM and ECR. MEP amplitude will be defined as the peak-to-peak voltage, i.e., the difference between the maximum and minimum values of the EMG signal following each TMS pulse. The onset latency will be calculated as the first post-stimulus time point at which the EMG signal exceeds 2 standard deviations above the baseline activity (computed from the 100 ms pre-stimulus window). Trials affected by pre-activation or noise will be excluded. The RMT will be calculated for each muscle as the lowest stimulator intensity able to evoke MEPs ≥ 50 μV in at least 5 out of 10 trials. RMT values will be expressed as a percentage of maximum stimulator output (MSO).

MEP amplitude, latency, and RMT will be analyzed using a mixed ANOVA, with Medication State (ON vs. OFF) and Muscle (SbM vs. ECR) as within-subjects factors, and Group (PD vs. Controls) as a between-subjects factor. Post-hoc comparisons will be corrected using Bonferroni adjustments. In case assumptions of normality or sphericity are violated, appropriate corrections or non-parametric counterparts (e.g., Wilcoxon signed-rank test or Mann–Whitney U test) will be applied. In addition, correlational analyses (Pearson or Spearman, depending on distribution) will be conducted to explore the relationships between neurophysiological measures (MEP amplitude, latency, RMT) and clinical variables such as fatigue and mood symptoms, as measured by the Parkinson’s Disease Fatigue Scale (PFS-16) and the Hospital Anxiety and Depression Scale (HADS).

##### 2.2.3.1 Expected results

We expect that the excitability of the CB tract, as measured by MEPs recorded from the SbM, will be significantly modulated by dopaminergic state in PD patients. In particular, we anticipate that MEP amplitude will be increased in the ON-medication condition compared to the OFF condition, reflecting enhanced motor cortical output following levodopa administration. This prediction is consistent with previous studies showing that dopaminergic treatment can increase MEP amplitude in PD, particularly in circuits sensitive to motivational and affective modulation. While both submental and control muscles (ECR) are expected to show some degree of responsiveness to motor cortical stimulation, we hypothesize that dopaminergic modulation will be more pronounced in the target muscle (SbM), due to the CB system’s closer integration with limbic and dopaminergic reward pathways. This is supported by evidence from animal models, where unilateral dopaminergic lesions lead to impairments in tongue force and rhythm (Skitek et al., 1999), and by neurophysiological models implicating dopamine in bulbar motor control (Stanford et al., 2003).

Moreover, we expect a significant reduction in RMT for the SbM in the ON-medication condition, indicating lower cortical excitability thresholds under dopaminergic facilitation. Similarly, MEP onset latency may decrease when patients are ON medication, reflecting more efficient synaptic transmission and conduction time in corticobulbar pathways.

Taken together, the results are expected to support the hypothesis that CB excitability is sensitive to dopaminergic modulation and may serve as a non-invasive physiological marker of central motor and motivational system function in PD.

## 3 Discussion

This research protocol, integrating two complementary studies on heathy individuals and in patients with PD, seeks to address a critical gap in understanding the role of the CB tract in motivational appetitive arousal and its potential as a biomarker for dopaminergic dysfunction. By integrating state-of-the-art neurophysiological tools with innovative experimental paradigms, the study aims to establish by combining spontaneous SbM activity (Study 1) and TMS-elicited MEPs (Study 2), this protocol investigates CB excitability as a clinically relevant physiological indicator of CB excitability and reward-related processing.

Consistent with the preregistered nature of the study, all hypotheses are framed in exploratory terms. While prior evidence suggests that corticobulbar excitability may be sensitive to motivationally salient states, the current protocol does not assume a causal role for the corticobulbar tract in reward processing. Rather, we aim to investigate whether it can serve as a physiological correlate of motivational arousal, particularly in the context of dopaminergic modulation.

Study 1 investigates the relationship between SbM activity and preferences in healthy participants using a VR shopping task. The incorporation of VR technology is a key innovation, providing an ecologically valid platform to explore the physiological correlates of decision-making in a controlled yet realistic environment (Lucifora et al., 2024; Nucera, 2024; Kurakoc et al., 2025). Prior research highlights the close connection between SbM activity and the reward-related processing, particularly its role in evaluating pleasant and unpleasant stimuli (Vicario et al., 2014, 2017; Ferraioli and Vicario, 2024). Building on previous suggestions (Ferraioli and Vicario, 2024), we hypothesize that increased SbM activity will correlate with higher ratings of product preference and purchase likelihood, and could inform predictive preference models, offering novel insights into reward-driven behaviors. The immersive nature of VR further enhances ecological validity, enabling a nuanced exploration of the interplay between physiological responses and behavioral preferences.

Study 2 extends these findings to a clinical population, investigating the effects of dopaminergic modulation on CB excitability in patients with PD. By measuring MEPs elicited through single-pulse TMS protocol, this study provides a direct assessment of CB excitability through SbM motor area. The inclusion of “off-medication” and “on-medication” conditions enables a detailed analysis of Levodopa’s effects on CB tract function. This approach aligns with prior evidence demonstrating the dopaminergic system’s critical role in regulating motivational, affective and motor processes (Bressan and Crippa, 2005; Marsden, 2006; De Letter et al., 2007; Lees et al., 2009; Baik, 2013; Speranza et al., 2021). Importantly, this study also compares MEPs from the SbM and a control muscle, ensuring specificity in assessing CB excitability.

### 3.1 Methodological considerations and limitations

Swallowing dysfunction in PD has been addressed through various interventions, including deep brain stimulation (DBS) and pharmacological treatment with levodopa. DBS has shown beneficial effects on bulbar symptoms in some patients (Chang et al., 2021b), although its use remains limited to clinical applications due to its invasiveness. On the other hand, the efficacy of levodopa in improving swallowing function appears inconsistent across studies, possibly reflecting inter-individual variability in bulbar circuit responsiveness (Chang et al., 2021a). These findings underscore the complexity of investigating corticobulbar involvement in PD and highlight the need for non-invasive tools capable of probing this pathway with functional resolution.

In this context, TMS offers a valuable method for assessing CB excitability at the cortical level, combining high temporal resolution, focal stimulation, and a well-established safety profile (Lefaucheur et al., 2020; Rossi et al., 2021). It enables precise, time-locked stimulation of motor cortical regions such as tM1 area, allowing for the controlled investigation of descending motor outputs through corticobulbar projections (Nardone et al., 2015; Spampinato et al., 2023). This makes TMS-elicited MEPs particularly well-suited for experimental protocols aimed at capturing the dynamic interaction between motivational states, pharmacological interventions, and bulbar motor excitability. Importantly, we intend to use a figure-8 coil to ensure high focality during stimulation. This approach is crucial to avoid confounding factors that can arise from stimulating large portions of the motor cortex, which can happen with less focal coils.

Moreover, while the study is methodologically well-grounded, it is important to acknowledge that the sample size in Study 2 is limited. Although a formal *a priori* power analysis (*α* = 0.05, 1 − *β* = 0.80) suggested a required sample of approximately 52 participants to detect a large effect size (Cohen’s *f* = 0.40), practical constraints in recruiting PD patients eligible for OFF-medication testing led us to plan enrollment of a reduced pilot sample of 30 participants. This choice reflects the exploratory nature of the current protocol and serves the primary goal of estimating effect sizes and assessing feasibility, to inform future confirmatory trials with adequate statistical power. Moreover, the study does not investigate the long-term effects of levodopa treatment beyond the three-month test–retest period. Finally, in Study 1, variability in VR immersion and susceptibility to motion sickness may influence the results. However, these concerns are mitigated through careful participant screening and the use of validated questionnaires.

### 3.2 Clinical relevance and future perspectives

This research carries significant implications for both basic and translational neuroscience. In healthy individuals, identifying physiological markers of hedonic preferences can advance our understanding of reward processing and decision-making theories. In PD, the study provides a non-invasive tool to monitor disease progression and assess treatment efficacy. In addition, by examining both spontaneous and evoked muscular responses along the same motor efference system, the protocol introduces a novel framework to assess motivational markers that bridge neurophysiological, pharmacological, and behavioral domains. Such a multimodal approach may inform the development of personalized therapeutic strategies in conditions characterized by motivational and dopaminergic dysregulation.

Together, the insights gained from these studies could drive the development of innovative diagnostic and therapeutic strategies, highlighting the importance of individualized approaches in managing dopaminergic dysfunction.

In conclusion, the proposed study not only aims to fill a key gap in the understanding of CB excitability and motivational function but also sets the stage for future research applying neurophysiological markers to clinically relevant questions in neurology and psychiatry.

## 4 Ethics and dissemination

This research protocol adheres to the highest ethical standards, ensuring participant safety, autonomy, and well-being while maintaining scientific rigor. Ethical approval has been obtained from the appropriate committees, and the study will comply with the Declaration of Helsinki (World Medical Association, 2013). Participants will be fully informed about the research objectives, procedures, and potential risks, with written informed consent obtained before enrollment. Measures are in place to protect participant privacy, including anonymizing identifiable data during analysis and dissemination.

To address potential risks, specific protocols will be implemented for each study. In Study 1, the use of VR technology, while immersive, may cause motion sickness or visual fatigue. Participants will be screened for susceptibility, and safety measures, including immediate access to medical support, will be available during experimental sessions. In Study 2, TMS application will follow international safety guidelines (Rossi et al., 2021), with pre-screening to exclude individuals with contraindications, such as epilepsy or metallic implants. PD patients, particularly during the “off-medication” condition, will be closely monitored to ensure their comfort and safety.

The dissemination strategy is designed to maximize the scientific and sociatal impact of this research (Massimino, 2022). Findings will be published in peer-reviewed journals, targeting both basic neuroscience and clinical audiences, and presented at international conferences to engage the broader scientific community. Collaborations with neurology clinics and research institutions will help translate findings into clinical practice, particularly for monitoring PD using SbM biomarkers.

By combining robust ethical oversight with a comprehensive dissemination plan, this research not only advances scientific understanding of corticobulbar excitability and dopaminergic function but also seeks to translate these insights into meaningful applications for improving patient care and public health.
